# Supplementary material for: Online learning success model for adults in open and distance education in Western China
Source: PLoS One. 2024 Feb 23;19(2):e0297515. doi: 10.1371/journal.pone.0297515 (PMC10890717; doi:10.1371/journal.pone.0297515)
Supplement: S1 Appendix — (DOCX) [file pone.0297515.s002.docx]

**Appendix** **1. The items with their sources**

| Measure | Code | Related studies |
| --- | --- | --- |
| **Information Quality** |  |  |
| 1. The information provided by the open distance learning system is useful | IQ1 | (Duckworth & Quinn, 2009) |
| 2.The information provided by the open distance learning system is clear and understandable. | IQ2 |  |
| 3. The information provided by the open distance learning system is up-to-date. | IQ3 |  |
| 4. The information provided by the open distance learning system is adequate. | IQ4 |  |
| **System quality** |  |  |
| 1.The open distance learning system offers flexibility of time and place for learning. | SQ1 | (Urbach et al., 2010) |
| 2.The open distance learning system offers multimedia courses. | SQ2 |  |
| 3.The open distance learning system is convenient and well-structured. | SQ3 |  |
| 4.The open distance learning system enables interactive communication between lecturers and students. | SQ4 |  |
| **Service Quality** |  |  |
| 1.The open distance learning system support service provides prompt service. | SEQ1 | (Urbach et al., 2010) |
| 2.The open distance learning system support service has convenient operating hours. | SEQ2 |  |
| 3.The open distance learning system support service is reliable. | SEQ3 |  |
| 4.The open distance learning system support service is convenient to communicate. | SEQ4 |  |
| **TPACK** |  |  |
| **TK** |  |  |
| 1. I think my lecturer has the technical skills to use computers effectively. | TK1 | (Valtonen et al., 2017; Wang, 2019) |
| 2.I think my lecturer know how to solve his/her own technical problems when using technology. | TK2 |  |
| 3.I think my lecturer keeps up with important new technology. | TK3 |  |
| 4.I think my lecturer can use social media (e.g. QQ, Tik Tok, and Wechat) | TK4 |  |
| **PCK** |  |  |
| 1.I think my lecturer can choose technologies that enhance the teaching approaches for a lesson. | PCK1 | (Valtonen et al., 2017; Schmid et al., 2009) |
| 2.I think my lecturer can choose technologies that enhance students’ learning for a lesson. | PCK2 |  |
| 3.I think my lecturer can adapt the use of the technologies that I am learning about to different teaching activities. | PCK3 |  |
| 4. I think my lecturer can think critically about how to use technology in his/her classroom. | PCK4 |  |
| 5.I think my lecturer can know how to guide students in planning their own learning in the subject he/she is teaching. | PCK5 |  |
| **TCK** |  |  |
| 1. I think my lecturer can use software created specifically for his/her teaching subject. | TCK1 | (Valtonen et al., 2017; Schmid et al., 2009) |
| 2.I think my lecturer know about the technology that I have to use in order to learn the content of a teaching subject. | TCK2 |  |
| 3.I think my lecturer can use appropriate technology (e.g. multimedia resources) to represent the content of his/her teaching subject. | TCK3 |  |
| 4. I think my lecturer know which new technologies are currently being developed in the field of his/her subject. | TCK4 |  |
| **TPACK** |  |  |
| 1.I think my teacher can use technology to determine students’ needs related to a content area in the pre-teaching process. | TPACK1 | (Santos & Castro, 2021; Schmid et al., 2009) |
| 2. I think my lecturer can formulate in-depth discussion topics about the content knowledge and facilitate students' online collaboration with appropriate tools (e.g. Google Sites). | TPACK2 |  |
| 3. I think my teacher can implement effective classroom management in the teaching and learning process in which technology is used. | TPACK3 |  |
| 4.I think my lecturer can design lessons that integrate content, technology and pedagogy appropriately for student-centered learning. | TPACK4 |  |
| 5. I think my teacher can cooperate with other disciplines regarding the use of technology to solve problems encountered in the process of presenting content. | TPACK5 |  |
| **Continuous Use** |  |  |
| 1.I continue using the online learning system to Retrieve information. | US1 | (Urbach et al., 2010) |
| 2. I continue using the technology of online learning system. | US2 |  |
| 3. I continue using the online learning system to communicate with colleagues and teachers. | US3 |  |
| 4. I continue using the online learning system to publish information | US4 |  |
| 5. I continue using the online learning system to execute courses' tasks. | US5 |  |
| **User Satisfaction** |  |  |
| 1.I am satisfied with the performance of the open distance learning system | US1 | (Arbaugh, 2000; Hassanzadeh et al., 2012) |
| 2.I enjoy using the open distance learning system in my study | US2 | (Arbaugh, 2000) |
| 3.The open distance learning system satisfies my educational needs | US3 | (Hassanzadeh et al., 2012) |
| 4.Overall, I am pleased with the experience of using the open distance learning system | US4 | (Cidral et al., 2018) |
| **Benefits** |  |  |
| **Academic development** |  |  |
| After using online learning system, I am able to perform these tasks: 1. Comprehend course content. | NB1 | (Hébert & Hauf, 2015) |
| 2. Understand career interests. | NB2 |  |
| 3. Reflect on my experience and learning. | NB3 |  |
| 4. Apply course content outside of the classroom. | NB4 |  |
| 5. Understand how the subject matter of the course can be used in everyday life. | NB5 |  |
| **Career development** |  |  |
| 6. The courses can guide my practical work. | NB6 | (Orpen, 1994) |
| 7. The course help me to determine goals for my career throughout my lifetime. | NB7 |  |
| 8. The course give me a lot of thought to plan and scheme for my career goals. | NB8 |  |
| 9. The course is very important to me for achieving my career goals. | NB9 |  |
| 10. After the course study I can help my superiors to complete important tasks. | NB10 |  |
| 11.After the course study I seek opportunities actively. | NB11 |  |
| **Personal development** |  |  |
| 12. My capability to identify and resolve problems has been improved | NB12 | (Chen & Huang, 2017) |
| 13.My prior frustration has been well responded. | NB13 |  |
| 14.My time management skills have been improved | NB14 |  |
| 15.My perspective of value has been changed | NB15 |  |
| 16.My confidence has been increased | NB16 |  |
